# Different analgesic effects of adenosine between postoperative and neuropathic pain

**DOI:** 10.1007/s00776-012-0302-0

**Published:** 2012-09-21

**Authors:** Gotaro Yamaoka, Hideki Horiuchi, Tadao Morino, Hiromasa Miura, Tadanori Ogata

**Affiliations:** 1 Spine Center, Ehime University Hospital, Tohon, Ehime 791-0295 Japan; 2 Department of Bone and Joint Surgery, Ehime University Graduate School of Medicine, Tohon, Ehime 791-0295 Japan

## Abstract

**Background:**

Adenosine is an endogenous neuromodulator in both the peripheral and central nervous systems. Adenosine inhibits pain signals by hyperpolarizing neuronal membrane.

**Methods:**

To clarify the effects of adenosine on pain signals, we tested intrathecal adenosine injection in two neuropathic pains (spinal cord compression and chronic constriction of sciatic nerve) and postoperative pain (plantar incision).

**Results:**

In all three kinds of pain models, significant shortening of withdrawal latencies to thermal stimulation were detected from 24 h to 1 week after the surgery. Significant improvements of pain sensation were observed in all three models after intrathecal injection of Cl-adenosine 24 h after surgery. At 72 h after surgery, intrathecal Cl-adenosine injection inhibited hyperalgesia in the two neuropathic pain models but not in the postoperative pain model. Adenosine A1R messenger RNA (mRNA) expression significantly decreased in the plantar incision model. Adenosine A1R protein levels also decreased compared with the other two models and normal control.

**Conclusions:**

These results suggest that adenosine effectively inhibits pain signals in neuropathic pain but is less effective in postoperative pain because of the decrease in adenosine A1 receptors.

## Introduction

A large number of patients suffer from sciatica, low back, and postoperative pain, treating which is one of the most important topics for clinicians in orthopedic surgery or anesthesiology. However, knowledge about pain signals remains insufficient. Nonsteroidal anti-inflammatory drugs (NSAIDs) have been commonly used but are effective only for treating inflammatory pain by inhibiting prostaglandin production. Understanding endogenous pain modulatory systems that alter nociceptive transmission in the nervous system may lead to the development of pain treatment. The opioid receptor (OR) and monoamine systems are the main inhibitory transmission mechanisms against pain sensation. Endogenous opioid receptor agonists are very powerful analgesic mechanisms. At the spinal cord level, delta opioid receptors play an important role in antinociception [1]. Clinically, μOR agonists, such as morphine, are commonly used for treating rather serious pain, such as postoperative pain. Monoamines, such as noradrenalin [2] and serotonin [3], are also strong endogenous pain-relieving agents. Monoamines inhibit pain signals by the activating γ-aminobutyric acid (GABA) signals [4]. Increasing monoamines with reuptake inhibitors has been used for pain relief in patients with several neuropathic pains [5]. We previously reported that serotonin reuptake inhibitors ameliorated neuropathic pain induced by spinal cord injury [6]. In the study reported here, we focused on adenosine receptors, another endogenous nerve transmission system. Adenosine is an endogenous neuromodulator that inhibits synaptic transmission in both the peripheral and central nervous systems [7]. Adenosine A1 receptor activation can produce postsynaptic inhibition by activating potassium (K^+^) channels [8]. The working mechanism of adenosine A1 receptors on pain signals is believed to inhibit pain signals by hyperpolarization of the neuronal membrane. Mice lacking adenosine A1 receptors exhibited increased nociceptive responses [9, 10]. These reports indicate that adenosine A1 receptor signal activation may become a new therapeutic method for treating several kinds of pain. We previously reported that selective adenosine A1 receptor agonists [R-PIA: R(-)N6-(2phenylisopropyl) adenosine] inhibited thermal hyperalgesia after spinal cord injuries in rats [11]. To establish a clinical use for adenosine, we examined which kinds of pain are the ideal targets for adenosine treatment and the best timing for adenosine application.

In the study reported here, to clarify the effects of adenosine on pain signals, we tested intrathecal adenosine injections in several kinds of animal pain models. In addition, change of adenosine receptors in the spinal cord was assessed by real-time polymerase chain reaction (PCR) and immunohistochemistry.

## Materials and methods

### Animals

One hundred and one female Wistar rats (250 g, purchased from Japan Clea Co., Japan) were used for this study. Experiments were performed according to the ethical recommendations of the Committee for Research and Ethical Issues of the International Association for the Study of Pain [12]. The research protocol was accepted by the Ethical Committee for Animal Experiments of Ehime University (Ehime, Japan).

### Plantar incision model (PI model)

A plantar incision (PI) was made similar to that described by Brennan et al. [13]. A 1-cm longitudinal incision through skin, fascia, and muscle was made in the left hind paw. The skin was closed with two 4-0 nylon sutures.

### Spinal cord mild-compression injury model (SCI model)

The spinal cord compression model was produced according to our previous reports [6, 11]. Under general anesthesia with halothane, the rat spinal cord was carefully exposed by removing the vertebral lamina at the 11th vertebra. Direct compression was performed using a 20-g weight, of which the point of contact to the dura consisted of very soft and rounded silicone. The weight was gently placed on the thoracic spinal cord extradurally for 20 min (SCI). We observed no serious damage, such as hyperextension, paresis of the hind limbs, or histological hemorrhage with tissue destruction at the point of compression. In some experiments, a laminectomy of the 11th vertebra was performed without SCI (sham).

### Chronic constriction injury of sciatic nerve model (CCI model)

Our CCI model was produced according to our previous report [14]. Under general anesthesia with halothane, the sciatic nerve was carefully exposed at the middle of the femur. Chronic constriction was produced by ligation in the left side only. A 27-gauge needle was placed along the sciatic nerve and the nerve and needle were ligated using polyglycolic acid strings (4-0, Nesco Company, Osaka, Japan) at four points. The intervals between constriction points were 1 mm. Too much constriction produces irreversible nerve damage; therefore, careful constriction is required to produce the appropriate level of damage. After the strings were inserted, the needle was removed. The strings expand as they absorb moisture after being placed in the animals. Therefore, constriction slowly increases after the operation. Usually, stable hypersensitivity, such as allodynia and hyperalgesia, was observed 3 days after operation. Animals that did not successfully reveal allodynia and hyperalgesia were excluded from the study.

### Intrathecal application of Cl-adenosine

Cl-adenosine (10 nmol in 10 μl; Research Biochemicals Inc. MA,USA), a nonselective adenosine receptor agonist, was intrathecally injected through the intervertebral foramen between L3 and L4 at 24 and 72 h after surgery. In each model, animals injected with 10 μl of saline instead of Cl-adenosine solution were defined as vehicles.

### Evaluation of thermal hyperalgesia

To evaluate the withdrawal threshold of thermal paw stimulation, we used the Hargreaves’ plantar test apparatus (Ugo Basile, Varese, Italy). Rats were placed on a 2-mm-thick glass floor. A mobile infrared heat generator with an aperture of 10 mm diameter was aimed at the rat’s hind paw from under the floor. When the rats felt pain and withdrew their paw, the power was shut off and the reaction time (paw withdrawal latency) was recorded automatically. Shortening withdrawal latency indicated thermal hyperalgesia. The experiments were repeated three times, at 5-min intervals, on each paw. Averages of measurements taken were used as data. The temperature of the glass floor was kept at 22.5–23.5 °C. 

### Real-time PCR analysis for adenosine A1R mRNA expression

In the CCI and the PI models, L4 and L5 lumbar spinal segments were taken for messenger RNA (mRNA) measurement. In the SCI model, 1 cm of the cord at the center of the compressed part was taken. Spinal cord tissue was dissected under RNase-free conditions, and samples were stored at −80 °C before use. Total RNA was extracted by tissue homogenization in Trizol reagent, quantified by absorbance at 260 nm, normalized, and reverse-transcribed into first-strand complementary DNA (cDNA) using an RNeasy mini kit (QIAGEN, MD, USA). For the* A1R* gene (TaKaRa. Shiga, JAPAN), the forward primer was 5′-ATCGATACCTCCGAGTCAAGATCC-3′ and the reverse primer was 5′-TCCAGTCTTGCTCTACCACACTCAG-3′. For the* GAPDH* gene (TaKaRa), the forward primer was 5′-GGCACAGTCAAGGCTGAGAATG-3′ and the reverse primer was 5′-ATGGTGGTGAAGACGCCAGTA-3′. Two-step real-time PCR denaturing, annealing, and extension reactions were performed for 40 cycles of 15 s at 95 °C and then 1 min at 60 °C (for* A1R* and* GAPDH*). Increasing curves of reporter dye fluorescence emission were recorded and analyzed with the SYBR^®^ Premix Ex Taq™ (TaKaRa) to determine the threshold cycle (Ct) value. Each sample was run and analyzed in triplicate, and Ct values for* A1R* were subtracted from Ct values of* GAPDH* to yield ΔCt values. The average ΔCt was calculated for the control group, and this value was subtracted from the ΔCt of all other samples (including the control group). This resulted in a ΔΔCt value for all samples, which was then used to calculate the fold induction of the mRNA levels of* A1R* using the formula 2^−ΔΔCt^, as recommended by the manufacture (Bio-Rad, Hercules, CA, USA) [15, 16].

### Immunohistochemistry

Animals were sacrificed by deep anesthesia followed by decapitation. The spinal cord at the 11th vertebral level (SCI model) and L4–L5 lumbar segments (lumbar enlargement; CCI and PI models) were immediately removed, and axial freezing microtome sections of 10-μm thickness were prepared. The sections were fixed on glass slides with 4 % paraformaldehyde in phosphate-buffered saline (PBS) for 5 min. Then, after washing twice with PBS, slices on the slides were exposed to an anti-adenosine A1R antibody (Abcam, Inc. Cambridge, UK: 1 μg/ml in PBS) overnight at 4 °C. Slices were then washed twice with PBS and exposed to a fluorescein isothiocyanate (FITC)-conjugated anti-rabbit immunoglobulin G (IgG) antibody (SIGMA, MO, USA: 20 μg/ml in PBS) for 30 min. Sections were then observed under fluorescent microscopy.

### Data analysis

For statistical analysis, analysis of variance (ANOVA), followed by Fisher’s protected least significant difference (PLSD), was used.

## Results

**Fig. 1:**
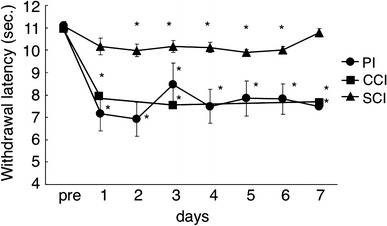
Time course of withdrawal latency by thermal stimulation following three kinds of pain models. After measurement of withdrawal latencies, rats received operations. Plantar incision (*PI*; *n* = 6), chronic constriction injury (*CCI*; *n* = 6), and spinal cord injury (*SCI*; *n* = 15) were performed. Measurements were taken every 24 h until the 7th day following operations. Data are the mean ± standard error of mean (SEM). Statistical significance compared with preoperation levels for each time point is represented with an *asterisk* (*P < 0.05)

In all three kinds of pain models, significant shortening of withdrawal latencies were detected from 24 h to 1 week after the surgery (Fig. 1). Hyperalgesia was seen only ipsilaterally to the surgery side in the PI and CCI models. In the foot contralateral to the operation, withdrawal latencies against thermal stimulation was identical to that in normal animals (11.1 ± 0.24 s).

**Fig. 2:**
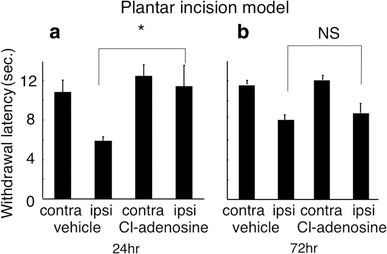
Effects of Cl-adenosine on thermal stimulation in the plantar incision model at 24 h (**a**) and 72 h (**b**) after surgery. Data are mean ± standard error of mean (SEM) (*n* = 6 in each time point). *Asterisk* statistical significance (*P < 0.05)

**Fig. 3:**
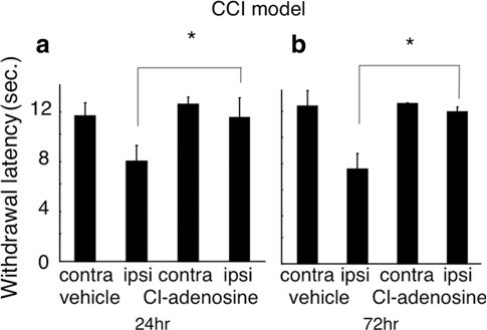
Effects of Cl-adenosine on thermal stimulation in the chronic constriction injury model at 24 h (**a**) and 72 h (**b**) after surgery. Data are mean ± standard error of mean (SEM) (*n* = 6 in each time point). *Asterisk* statistical significance (*P < 0.05)

**Fig. 4:**
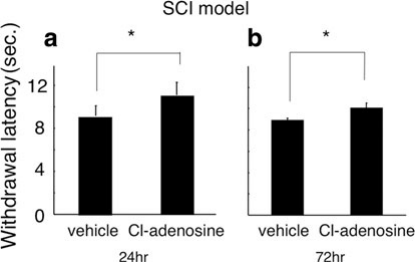
Effects of Cl-adenosine on thermal stimulation in the spinal cord injury model at 24 h (**a**) and 72 h (**b**) after surgery. Data are mean ± standard error of mean (SEM) (*n* = 6 in each time points). *Asterisk*, statistical significance compared vehicle animal (*P < 0.05)

In the postsurgical model (PI model; Fig. 2), apparent thermal hyperalgesia was seen in the foot ipsilateral to the PI in the vehicle animal both at 24 and 72 h after the operation. When Cl-adenosine (10 nmol in 10 μl) was applied 24 h after surgery, pain threshold returned to normal (*P* < 0.05, 11.37 ± 0.97 s). However, the antihyperalgesic action of Cl-adenosine was not seen when the injection was done 72 h after the operation. In the CCI model (Fig. 3), hyperalgesia was also seen in the foot ipsilateral to sciatic nerve constriction in the vehicle animal both at 24 and 72 h after the operation. Intrathecal injection of Cl-adenosine normalized withdrawal threshold against thermal stimulation at 24 (11.36 ± 0.69 s) and 72 h (12.07 ± 0.19 s) after the operation. The difference of the Cl-adenosine effect in the PI and CCI models was seen at 72 h after the operation. In the SCI model (Fig. 4), thermal threshold decreased both at 24 and 72 h after the operation compared with that in the sham-operated animals. Intrathecal injection of Cl-adenosine significantly (*P* < 0.05) increased withdrawal latency both at 24 (11.12 ± 0.55 s) and 72 h (10.47 ± 0.67 s) after the operation.

**Fig. 5:**
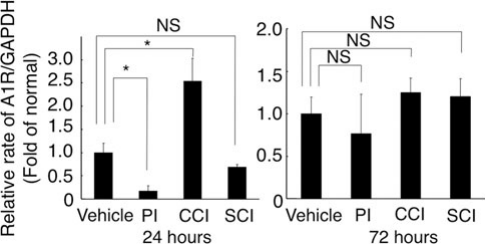
Comparison of adenosine A1R messenger RNA (mRNA) expression by real-time polymerase chain reaction (PCR) analysis in the normal group and the three pain models. Data are mean ± standard error of mean (SEM) (*n* = 3 in each column). *Asterisk* statistical significance compared with normal group (*P < 0.05)

Real-time PCR analysis was represented as folds to normal mRNA content (Fig. 5). Twenty-four hours after operation, adenosine A1R mRNA expression significantly (*P* < 0.05) decreased in the PI model (0.2 folds compared with the normal level). In the SCI model, adenosine A1R mRNA level decreased to 0.7 folds. On the other hand, in the CCI model it remarkably increased (2.5 folds), at 72 h after operation in all three models it moved toward the normal level. However, in the PI model, it was still lower than normal (0.7 folds).

**Fig. 6:**
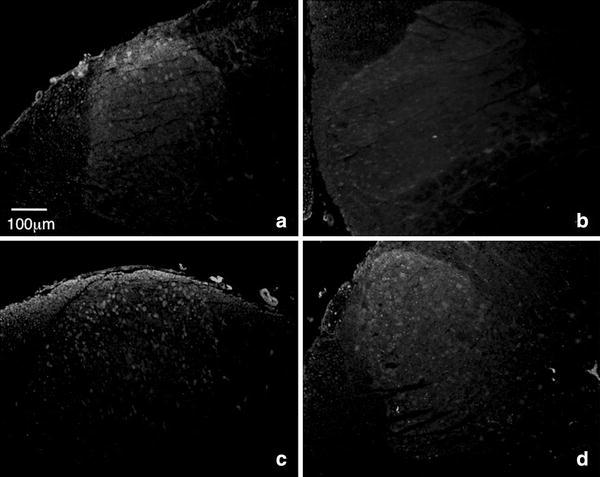
Adenosine A1R protein expression in spinal cord dorsal horn. Seventy-two hours after operation, spinal cord sections from the 11th vertebra of the spinal cord mild-compression (SCI) model and L4–L5 lumbar segment [lumbar enlargement; plantar incision (PI) and chronic constriction injury of sciatic nerve model (CCI) models and normal rat] were stained by anti-adenosine A1R antibody. Normal rat (**a**), PI model (**b**), CCI model (**c**), SCI model (**d**). Apparent decrease in adenosine A1R expression was observed in PI model

Adenosine A1R protein expression was evaluated immunohistochemically. In the normal animals, the A1 receptor was expressed mainly in lamina II of the dorsal horn. Twenty-four hours after operation, A1 receptors were maintained in all three models (data not shown). Three days after operation, A1 receptors were rather increased in the dorsal horn of the CCI model. A1 receptor levels in the SCI model were similar to that in the normal animal. On the other hand, A1 receptor proteins remarkably decreased in PI models 3 days after operation compared to the other two models and normal control (Fig. 6).

## Discussion

The dorsal horn of the spinal cord is believed to be one of the most important areas for pain signal transmission. In the spinal cord, adenosine A1 receptors are mainly distributed in postsynaptic neuronal-cells bodies and processes in dorsal superficial layers (lamina II) [17]. We also found that dense adenosine A1 receptor proteins existed in lamina II of the dorsal horn (Fig. 6). This area is not only for adenosine signal transmission but is also the main area of adenosine production. Ecto-5′-nucleotidase is a membrane-anchored protein that hydrolyzes extracellular adenosine 5′-monophosphate (AMP) to adenosine. Ecto-5′-nucleotidase is located on nociceptive neurons in dorsal root ganglia and on axon terminals in lamina II (substantia gelatinosa) of the spinal cord [18]. Thus, this area should be a main spot of endogenous pain relief by adenosine signaling.

We found the adenosine A1 receptor mRNA remarkably decreased in the spinal cord after PI but increased after sciatic nerve constriction (Fig. 5). This suggests that differential working mechanisms of adenosine in pain modulation existed in postoperative and neuropathic pain conditions. It is not clear why adenosine A1 receptor expression in the spinal cord dorsal horn was enhanced in our CCI models. A remarkable difference between the CCI and PI models is microglia activation in the spinal cord. Microglia proliferation and activation occurs in the spinal cord in the CCI model [19]. On the other hand, microglial activation was not evident following PI [20]. The activated microglia in the spinal cord may enhance the pain signal by releasing nitric oxide or cytokines. Meanwhile, adenosine A1 receptor activation counteracts microglia proliferation and activation after CNS injury [21]. Therefore, it is possible that adenosine A1 receptor mRNA expression enhanced to counteract the effect of activated microglia in our CCI model.

The change in adenosine A1 receptor mRNA expression of the SCI model was not remarkable compared with that in the CCI model (Fig. 5). Adenosine receptor was comparatively maintained in the mild SCI model. Although activated microglia also enhanced adenosine A1 receptor production in SCI model [22], direct ischemic damage might scale back the amount of A1 receptors. The pathological pain mechanism of the SCI model is different from that in the CCI model. SCI-induced pain is reported to induce inhibition of tonic descending inhibitory mechanisms, such as serotonergic and noradrenergic signaling, in the spinal cord [6]. Therefore, the working mechanisms of injected Cl-adenosine were different between CCI and SCI models. In the SCI model, injected Cl-adenosine may compensate damaged descending inhibitory signaling from brain to spinal cord. In the CCI model, in contrast, sensitivity to adenosine was increased in the dorsal horn by augmented adenosine A1 receptor expression.

The reason for decreased adenosine A1 receptor expression in our PI model also remains unclear. Hippocampal A1 receptor immunoreactivity in rats with cortex-kindled seizures remarkably decreased compared with that in normal rats [23]. The number of A1 receptors in the hippocampus was also decreased in aged rats compared with young adult rats [24]. In aged rats, adenosine production is activated by the increase of ecto-5′-nucleotidase. In addition, the amounts of adenosine in the synaptic cleft further increased due to the decrease in adenosine transporter activity [24]. It is possible that repetitive overstimulation of increased extracellular adenosine may induce adenosine A1 receptor expression inhibition in these models. In the PI pain model, stimulation from the hind paw may accelerate adenosine triphosphate (ATP) release from the afferent nerve terminals in the dorsal horn. The released ATP may be rapidly changed to adenosine by abundantly existing ecto-5′-nucleotidase. Then, adenosine A1 receptor mRNA expression may be depressed by overstimulation of adenosine in the same manner as in aged or kindled rat. Released ATP, which is the source of extracellular adenosine, may be depleted with time; however, adenosine A1 receptor expression inhibition continued for several days after PI. 

As adenosine is a common substance existing in the entire nervous system, it inhibits nerve transduction at the baseline level. When the adenosine A1 receptor was blocked at the spinal cord level, the threshold of thermal pain decreased (heat hyperalgesia) [11]. Therefore, when adenosine A1 signal is reduced by decreasing adenosine A1 receptors, pain signals should be enhanced. If this phenomenon is a rational control of the self-protection system, A1 receptor inhibition may induce hyperalgesia as cautionary notices to avoid further nociception. The striking decrease of A1 receptor mRNA occurred 24 h after PI (Fig. 5), mRNA level recovered within another 2 days, and change in A1 receptor protein occurred later than that in mRNA level. Decreased A1 receptor protein was apparent 3 days after PI (Fig. 6). This indicates that desensitizing signals from adenosine are not effective for several days after incision. On the other hand, the desensitizing mechanism of adenosine signals was maintained after nerve injury. Therefore, although adenosine is a potential pain-relief agent, application of adenosine may not be effective in postoperative pain compared with that in neuropathic pain.

OR agonists have been used to treat several types of neuropathic or postoperative pain. However, several studies suggest that μOR agonists, like morphine, show decreased analgesic potency against neuropathic pain [25]. After peripheral nerve injuries, μOR expression significantly decreased ipsilateral to nerve injuries in the dorsal horn [26]. These reports suggest that μOR agonist application for treating neuropathic pain after a peripheral injury is not rational. Relatively large amounts of the agonist may be required to achieve a large enough analgesic effect, which may produce considerable side effects such as constipation or nausea/vomiting. These results suggest that adenosine may be a reasonable choice for treating neuropathic pain that has not responded well to the opioids.

Clinically, chronic neuropathic pain is one of the most serious pathological conditions in the field of orthopedic surgery. As a result of this study, we propose a new possible therapeutic module for treating neuropathic pain after peripheral nerve injuries. As most adenosine receptor agonists have a poor blood–brain barrier permeability [27], intrathecal injection of adenosine A1 receptor agonists, such as Cl-adenosine or R-PIA using a continuous infusion pump may effectively inhibit chronic neuropathic pain. Alternative ideas to increase the adenosine effect are to increase extracellular adenosine concentrations by amplifying production by Ecto-5′-nucleotidase or to inhibit adenosine uptake or degradation. Propentofylline, an adenosine reuptake inhibitor with a blood–brain barrier permeability, is reported to inhibit pain behavior after peripheral nerve injury in the rat [28]. Adenosine reuptake, kinase, and deaminase inhibitors may provide an avenue for the development of novel therapeutic methods against neuropathic pain.

## Conclusion

Our study indicates that intrathecally administered adenosine may be an effective pain-relief therapy for neuropathic pain but is not effective on postoperation pain. We also found a decrease in adenosine A1 receptors in the spinal cord dorsal horn of the PI model, which may explain the variation of adenosine effects among the three pain models.
